# Human Health Effects of Dichloromethane: Key Findings and Scientific Issues

**DOI:** 10.1289/ehp.1308030

**Published:** 2014-10-17

**Authors:** Paul M. Schlosser, Ambuja S. Bale, Catherine F. Gibbons, Amina Wilkins, Glinda S. Cooper

**Affiliations:** National Center for Environmental Assessment, U.S. Environmental Protection Agency, Washington, DC, USA

## Abstract

Background: The U.S. EPA’s Integrated Risk Information System (IRIS) completed an updated toxicological review of dichloromethane in November 2011.

Objectives: In this commentary we summarize key results and issues of this review, including exposure sources, identification of potential health effects, and updated physiologically based pharmacokinetic (PBPK) modeling.

Methods: We performed a comprehensive review of primary research studies and evaluation of PBPK models.

Discussion: Hepatotoxicity was observed in oral and inhalation exposure studies in several studies in animals; neurological effects were also identified as a potential area of concern. Dichloromethane was classified as likely to be carcinogenic in humans based primarily on evidence of carcinogenicity at two sites (liver and lung) in male and female B6C3F_1_ mice (inhalation exposure) and at one site (liver) in male B6C3F_1_ mice (drinking-water exposure). Recent epidemiologic studies of dichloromethane (seven studies of hematopoietic cancers published since 2000) provide additional data raising concerns about associations with non-Hodgkin lymphoma and multiple myeloma. Although there are gaps in the database for dichloromethane genotoxicity (i.e., DNA adduct formation and gene mutations in target tissues *in vivo*), the positive DNA damage assays correlated with tissue and/or species availability of functional glutathione *S*-transferase (GST) metabolic activity, the key activation pathway for dichloromethane-induced cancer. Innovations in the IRIS assessment include estimation of cancer risk specifically for a presumed sensitive genotype (GST-theta-1+/+), and PBPK modeling accounting for human physiological distributions based on the expected distribution for all individuals 6 months to 80 years of age.

Conclusion: The 2011 IRIS assessment of dichloromethane provides insights into the toxicity of a commonly used solvent.

Citation: Schlosser PM, Bale AS, Gibbons CF, Wilkins A, Cooper GS. 2015. Human health effects of dichloromethane: key findings and scientific issues. Environ Health Perspect 123:114–119; http://dx.doi.org/10.1289/ehp.1308030

## Introduction

In 2011, the Integrated Risk Information System (IRIS) of the U.S. Environmental Protection Agency (EPA) released an updated *Toxicological Review of Dichloromethane (Methylene Chloride)* (U.S. EPA 2011). In this commentary, we summarize exposure sources and the major findings, advancements, critical issues, and future research needs identified in the IRIS assessment, particularly with respect to the physiologically based pharmacokinetic (PBPK) model structure, approaches used to incorporate sensitivity based on genotype, and other sources of variability in the human population.

Dichloromethane [CASRN (Chemical Abstract Services Registry Number) 75-09-2] (or methylene chloride) is an organic solvent that has been used extensively in industrial settings (e.g., as an extraction solvent and as a metal cleaner) and consumer products (e.g., paint removers). In 2012, according to the U.S. EPA’s Toxic Release Inventory (TRI) database (http://iaspub.epa.gov/triexplorer/tri_release.chemical), a total of 3.4 million pounds of dichloromethane was released into the environment in the United States (U.S. EPA 2015). Because of its volatility, it is found mostly in air, and the predominant exposure for the general population occurs from inhalation (primarily from industrial emissions and consumer product use). In 1996, the national average concentration of dichloromethane in outdoor air was 0.47 μg/m^3^ (U.S. EPA 2002). Indoor inhalation exposures can result from using consumer products containing dichloromethane such as adhesives, spray shoe polishes, paint and adhesive removers [Agency for Toxic Substances and Disease Registry (ATSDR) 2000], and building materials and furnishings [California Environmental Protection Agency (CalEPA) 2000]. Average indoor air concentrations collected from urban, suburban, and rural residences between 1990 (after the 1989 ban on hairspray) and 2005 ranged from 0.4 to 3.5 μg/m^3^ (Dawson and McAlary 2009). Concentrations of dichloromethane in food and water are small compared to concentrations in air; thus, oral exposures are low. Drinking-water mean concentrations are generally less than one part per billion (ppb), which is below the 5 ppb maximum contaminant level (MCL) (CalEPA 2000). Dichloromethane releases to drinking-water sources are estimated to range between 0.3 and 2.4% of total environmental releases, much lower than the 86–95% estimate for atmospheric releases, with releases to land accounting for 2–12% (CalEPA 2000). Dermal absorption of dichloromethane has been demonstrated in animals (McDougal et al. 1986) and in humans (Stewart and Dodd 1964), making this pathway another potential exposure pathway of concern, particularly in occupational settings without adequate protective gear and with improper use of consumer products (e.g., paint strippers). High indoor air concentrations of dichloromethane have been reported in occupational settings, where the largest numbers of workers are potentially exposed to the chemical during metal cleaning, industrial paint stripping, and tasks using ink solvents (ATSDR 2000).

Metabolism of dichloromethane involves two primary pathways (see Supplemental Material Figure S1): an oxidative CYP2E1 pathway that is predominant at low exposures, and a glutathione *S*-transferase (GST)–catalyzed pathway that results in the conjugation of dichloromethane to glutathione (GSH) (Gargas et al. 1986; Guengerich 1997). The first step in the CYP2E1 (cytochrome P450 2E1) pathway is the formation of formyl chloride, most (> 97%) of which is metabolized further to carbon monoxide (CO) (Watanabe and Guengerich 2006). The GST-catalyzed pathway results in the formation of a GSH conjugate that is eventually metabolized to carbon dioxide (CO_2_). The conjugation of dichloromethane to GSH results in formation of two reactive intermediates that have been proposed to be involved in dichloromethane toxicity, *S*-(chloromethyl) GSH and formaldehyde (Hashimi et al. 1994). Although both pathways are expected to operate at all exposures, the CYP pathway predominates at lower exposure concentrations.

## Methods

The toxicological review was developed according to the general risk assessment guidelines (National Research Council 1983, 1994). The literature search strategy was based on the CASRN (75-09-02) in addition to the common names dichloromethane and methylene chloride. The search was conducted in collaboration between the assessment team and a medical librarian. Primary, peer-reviewed literature identified through September 2011 was included; literature searches were conducted in several databases including Toxicology Literature Online (TOXLINE; http://toxnet.nlm.nih.gov/newtoxnet/toxline.htm), PubMed (http://www.ncbi.nlm.nih.gov/pubmed/), and the Developmental and Reproductive Toxicology/Environmental Teratology Information Center (DART/ETIC) through TOXNET (Toxicology Data Network; http://toxnet.nlm.nih.gov/). Primary source studies examining any type of toxicity (e.g., reproductive, hepatic, cancer) in experimental animals and humans, in addition to toxicokinetic and mechanistic data, were identified. Each set of studies pertaining to a type of outcome was then evaluated, considering the influence of exposure parameters (route, level, duration), population or test animal, specificity of effect measure, and other aspects of study methods (U.S. EPA 2011). The results of the search and evaluation process were reviewed by an external review panel as part of the assessment development process (see Appendix A in U.S. EPA 2011).

In addition, as described by Cooper et al. (2011), a more focused search for epidemiology cancer studies was conducted using PubMed (last accessed on 30 March 2011) and additional search strategies focusing on related solvents. Studies with dichloromethane-specific results were selected for review; studies reporting estimated risks based on general categories of solvents were excluded. Information pertaining to participant characteristics, exposure assessment methodology and exposure levels, outcome definition and data sources, and potential confounding was used in the interpretation and synthesis of the collection of the 18 identified epidemiological studies of cancer. Tables summarizing these studies can be found in the IRIS assessment (U.S. EPA 2011).

## Major Toxic Effects of Dichloromethane

*Hepatic toxicity*. Hepatic effects are commonly observed forms of toxicity following inhalation (Burek et al. 1984; Nitschke et al. 1988) or oral (Serota et al. 1986a) dichloromethane exposure in rodents. Changes observed in animals following dichloromethane exposure include liver foci/areas of alteration, hepatocyte vacuolation (vacuolation of lipids in the hepatocyte), fatty liver, and necrosis, with effects seen beginning at 500 ppm (Burek et al. 1984; Nitschke et al. 1988). Available human studies do not provide an adequate basis for evaluation of hepatic effects, particularly given the limitations of serological measures of hepatic damage (Ott et al. 1983; Soden 1993). The mode of action and the causative agent(s) (i.e., parent compound, specific metabolite) for these noncancer effects are not known. However, dose–response analysis indicates that the correlation with the animal toxicity (hepatic vacuolation) was better for the liver-specific rate of CYP metabolism than for parent concentration or rate of GST metabolism. The lack of mode-of-action information and correlation with CYP metabolism for hepatic vacuolation is in contrast with the mechanistic data and internal metric used for cancer (discussed in further detail below). The U.S. EPA (2011) assumed distinct mechanisms for distinct end points, with different dose metrics applying.

*Neurotoxicity*. Consistent with other chlorinated solvents such as trichloroethylene and tetrachloroethylene, exposure to dichloromethane results in decreased motor activity, impaired memory, and changes in responses to sensory stimuli in mice and rats (reviewed by Bale et al. 2011). These effects are similar to those of other solvents that have been more extensively studied, such as toluene. Results from experimental studies in humans indicate that acute neurobehavioral deficits—measured, for example, by psychomotor tasks, tests of hand–eye coordination, visual evoked response changes, and auditory vigilance—may occur at concentrations > 200 ppm with 4–8 hr of exposure (Bos et al. 2006; Putz et al. 1979). Fewer studies have examined neurological effects of chronic exposures, although there is some evidence of an increased prevalence of neurological symptoms among workers with average exposures of 75–100 ppm (Cherry et al. 1981) and of long-term effects on some neurological measures (i.e., possible detriments in attention and reaction time in complex tasks) in workers whose past exposures were in the 100–200 ppm range (Lash et al. 1991). The chronic exposure studies are limited in terms of sample size and power considerations. However, the study by Lash et al. (1991) is noteworthy because it evaluated neurotoxicity of aircraft maintenance workers examined a mean of 5 years after retirement, and it indicates effects lasting after cessation of exposure. Additional studies of the long-term neurotoxic effects of chronic exposure are warranted. A recent review of mechanistic data indicates that dichloromethane may act directly at specific sites in the brain such as ligand and voltage-gated ion channels; these pharmacodynamic modulations are similar to other chlorinated solvents including trichloroethylene and tetrachloroethylene (Bale et al. 2011). Changes in glutamate, γ-aminobutyric acid, dopamine, serotonin, acetylcholine, and other neurotransmitters have also been observed. Target areas include the caudate nucleus and hippocampus (associated with learning and memory) and the cerebellum (associated with motor activity and neuromuscular function). More comprehensive studies specifically designed to determine the mode of action for dichloromethane-induced impairment of neurological functions have not been conducted (Bale et al. 2011).

*Cancer*. The 2-year animal bioassay data for dichloromethane provide evidence of carcinogenicity at two sites (liver and lung) in male and female B6C3F_1_ mice with inhalation exposure [National Toxicology Program (NTP) 1986] and at one site in male B6C3F_1_ mice (liver) with drinking-water exposure (Hazleton Laboratories 1983; Serota et al. 1986b). Exposure to inhalation concentrations of 2,000 or 4,000 ppm dichloromethane produced increased incidences of lung and liver tumors in B6C3F_1_ mice, with statistically significant trends observed in males and females at both sites (trend *p*-values ranged from < 0.001 to 0.013) (NTP 1986). In the oral exposure study, Serota et al. (1986b) indicated that there was no dose-related trend and no significant pair-wise differences with the controls. The analysis underlying these conclusions was not presented, but the statistical results presented in the full study report (Hazleton Laboratories 1983) support the interpretation of these data as indicative of a marginal trend (trend *p*-value = 0.058) with statistically significant increases in three of the four dose groups (*p* = 0.071, 0.023, 0.019, and 0.036 for the 50, 125, 185, and 250 mg/kg/day dose groups, respectively).

Human studies have observed associations between occupational exposure to dichloromethane and increased risk for several specific cancers, including brain cancer, liver and biliary tract cancer, non-Hodgkin lymphoma, and multiple myeloma (Cooper et al. 2011). The available cohort studies do not provide an adequate basis for drawing conclusions because they are limited by low statistical power for the relatively rare cancers such as liver and brain cancer, limited information pertaining to classification of hematopoietic cancers (and use of mortality, rather than incidence data for these cancers), and, for some studies, missing work history data or inability to recreate an inception cohort (U.S. EPA 2011). The hematopoietic cancer case–control studies are a relatively recent addition to the epidemiologic database, with seven large studies using relatively detailed exposure assessment procedures published since 2000 (Barry et al. 2011; Costantini et al. 2008; Gold et al. 2011; Infante-Rivard et al. 2005; Miligi et al. 2006; Seidler et al. 2007; Wang et al. 2009). The exposure assessments used a structured interview format, obtaining a lifetime job history with details on tasks and materials, with some studies including supplemental job-specific and industry-specific modules designed to obtain more detailed information pertaining to specific solvents. The four studies of incident non-Hodgkin lymphoma or multiple myeloma observed associations with dichloromethane exposure (ever exposed or highest category of exposure; odds ratios between 1.5 and 2.2) (Gold et al. 2011; Miligi et al. 2006; Seidler et al. 2007; Wang et al. 2009); these results indicate that a focus on these particular cancers is warranted in future research. Variability in GST metabolism has not been examined in relation to cancer risk in the epidemiological studies, but an interaction between the TT genotype of CYP2E1 rs2076067 (functional significance unknown), dichloromethane exposure, and risk of non-Hodgkin lymphoma was seen in one study (Barry et al. 2011). Genetic variability in susceptibility to solvent exposure, specifically dichloromethane exposure, is another area of future research needs.

In contrast with hepatic vacuolation (a noncancer effect), mode-of-action data are available for dichloromethane-induced cancer. The database of dichloromethane-induced chromosomal instability and DNA damage supports a mutagenic mode of carcinogenic action for dichloromethane, with a primary role for the GST metabolic pathway in carcinogenesis. This evidence includes *a*) *in vivo* evidence of chromosomal aberrations and micronuclei in mouse lung and peripheral red blood cells, tissues with a high availability of GST, without concurrent cytotoxicity (Allen et al. 1990), along with negative findings in bone marrow cells that have comparatively low availability of GST (Allen et al. 1990; Gocke et al. 1981; Sheldon et al. 1987; Westbrook-Collins et al. 1990); *b*) *in vitro* evidence of micronuclei (Doherty et al. 1996) and sister chromatid exchanges (Olvera-Bello et al. 2010) in human cells, mutation and DNA breaks in Chinese hamster ovary cells with GST-competent mouse liver cytosol (Graves and Green 1996), and mutation in bacterial strains that possess GST metabolic activity (in strains lacking GST, results were negative unless transfected with a mammalian GST gene) (DeMarini et al. 1997; Graves et al. 1994a; Pegram et al. 1997; Thier et al. 1993); and *c*) *in vivo* and *in vitro* DNA damage indicator assays, including the comet and sister chromatid exchange assays, with positive results in mouse red blood cells, liver, and lung, but not in bone marrow, where there is limited availability of GST (Allen et al. 1990; Graves et al. 1994b, 1995; Sasaki et al. 1998; Westbrook-Collins et al. 1990). This target-tissue site specificity is a key consideration in the evaluation of the available data. *In vivo* mammalian studies demonstrate site-specific effects, including chromosomal aberrations (Allen et al. 1990), DNA–protein cross-links (Casanova et al. 1992, 1996), DNA SSBs (single-strand breaks) (Graves et al. 1994b, 1995), and sister chromatid exchanges (Allen et al. 1990) in liver and/or lung cells of B6C3F1 mice following acute inhalation exposure to concentrations that produce liver and lung tumors with chronic exposures, and DNA damage (detected by the comet assay) after dichloromethane exposure was enhanced in liver tissue, but not in sites with more limited GST metabolism (stomach, kidney, brain, or bone marrow) in CD-1 mice (Sasaki et al. 1998). Adding to the weight of evidence for a mutagenic mode of action for dichloromethane are the observations that most positive results, both *in vitro* and *in vivo*, occurred at noncytotoxic doses; that several studies observed an increased incidence of genotoxicity with dose; and that *in vivo* formation of DNA damage was observed within 24 hr of acute exposures (Graves and Green 1996; Graves et al. 1995; Hu et al. 2006; Sasaki et al. 1998).

The relevance of the GST-mediated pathway versus the CYP-mediated pathway for cancer, at least in the liver, is also strongly supported by the PBPK model predictions. Between inhalation exposures to 2,000 and 4,000 ppm there is a significant increase in the incidence of liver cancers (hepatocellular carcinomas or adenomas) in both male and female mice (Mennear et al. 1988; NTP 1986), but the PBPK model predicts nearly complete saturation of hepatic CYP metabolism by 2,000 ppm. For example, in female mice, liver tumor incidence increases from 35% to 87% between these exposure levels, but the CYP-mediated liver-specific dose increases only from 3.1 to 3.2 g metabolized/L liver tissue/day and the whole-body CYP dose from 132 to 136 mg metabolized/kg body weight/day. (This lack of correlation between CYP metabolism and cancer is clearly distinct from the good correlation seen between CYP metabolism and noncancer effects, described previously.) Application of these doses with the cancer responses results in unacceptable fits of the multistage cancer benchmark dose model.

The relevance of the bioassay studies of dichloromethane in mice to humans in low-exposure scenarios has been questioned based on the high exposure conditions of the genotoxicity studies and animal bioassays, the high background rates of liver cancer in male B6C3F_1_ mice, and the relatively high GST activity in mice (Green 1997). However, as described in the subsequent section, the variation in GST activity is directly incorporated into the PBPK modeling, and both pathways are expected to operate, even at low exposures. In addition, differences in the localization of GST-Theta (T)1 within cells, another source of interspecies variation, support the relevance of the mouse liver tumor bioassay data to humans. Localization is seen in the mouse nuclei of hepatocytes and bile-duct epithelium, but a preferential nuclear localization of GSTT1 is not seen in the rat (Mainwaring et al. 1996). In human liver tissue, however, nuclear localization of GSTT1 has been seen in some hepatocytes and in nuclei of bile duct epithelial cells (Sherratt et al. 2002).

## PBPK Modeling

Dichloromethane metabolism occurs primarily in the liver but also in lung tissues, particularly in mice. The PBPK model structure for dichloromethane traces back to one originally developed by Gargas et al. (1986) (see Supplemental Material, Figure S2). This basic structure has remained mostly unchanged through numerous revisions published since then (e.g., Andersen et al. 1987; David et al. 2006). The model version used includes tissue compartments for the liver, fat, and slowly and rapidly perfused tissue groups, with metabolism by two pathways representing oxidative metabolism primarily via CYP2E1 and conjugation via GST in both the liver and lung tissue compartments.

As described above, the best (most likely) estimator of cancer risk for dichloromethane continues to be GST-mediated metabolism, so confidence in human cancer risk predictions based on the PBPK model depends on its ability to accurately predict the fraction of CYP- versus GST-mediated metabolism. A feature of the internal dosimetry predicted by the PBPK model is that as exposure level increases and the CYP-mediated pathway becomes saturated, a higher fraction of dichloromethane is metabolized by the GST-mediated pathway. Viewed in the other direction, going from high to low exposure levels, as the CYP pathway becomes nonsaturated, the fraction metabolized by the GST pathway is attenuated. CYP and GST effectively compete for the available dichloromethane, and at low exposure levels CYP metabolism in humans is predicted to predominate. The PBPK model integrates the competition between the two pathways with the physiological determinants of internal dose to predict the nonlinear exposure–dose relationship for both pathways. At low exposures, the GST metabolic rate remains nonzero, has a positive slope versus exposure level, and hence is consistent with the standard assumption of low-dose linearity for carcinogens.

*Probabilistic modeling of the human population*. The human dichloromethane PBPK model by David et al. (2006) specifically used a probabilistic description of model parameters such as body weight, cardiac output, and metabolic rates to describe a distribution of dosimetry among humans. David et al. (2006) used Bayesian analysis to update prior (initial assumed) distributions for the various metabolic parameters to match a variety of human pharmacokinetic data, while also using but not updating distributions for physiological parameters, because the latter are not expected to be identifiable based on such data and are quite well known already. To validate the statistical results, the U.S. EPA (2011) reproduced the Bayesian analysis and obtained essentially identical posterior distributions for the fitted parameters. (Small differences are inevitable given the probabilistic methodology.)

David et al. (2006) expanded the variability for a key parameter in the human population from that estimated by Bayesian analysis for the study populations from which the pharmacokinetic data were derived. In particular, some individuals carry one or two null alleles for the key enzyme, GSTT1, and the frequency of the double-null (–/–) allele varies between 62% in Asian Americans and only 10% among Hispanics (Haber et al. 2002). The analysis conducted by David et al. (2006) and the U.S. EPA (2011) used the average frequency of the three genotypes (+/+, +/–, and –/–) based on the percentage of these ethnic groups in the United States and the frequency of the genotypes within each group. David et al. (2006) and the U.S. EPA (2011) then assumed a normal distribution for GSTT1 activity among GSTT1^+/+^ individuals derived from the results of Warholm et al. (1994) who measured GSTT1 activity in human red blood cells, with the mean activity being a fitted parameter. The distribution of activity among heterozygous (+/–) GSTT1 individuals was assumed to be one-half that of the +/+ population, and the activity was assumed to be zero in –/– individuals.

The physiological parameter distributions used by David et al. (2006), however, represent a segment of the adult population ranging from approximately 20 to 40 years of age, rather than the population as a whole. In addition, David et al. (2006) did not incorporate a more representative distribution of CYP2E1. Although CYP2E1 activity is not known to vary with ethnicity, its variability has been measured and described using data from tissue donors (Lipscomb et al. 2003). Therefore, we updated the human physiological distributions based on data from a number of sources of demographic, physiological, and metabolic data to describe the expected distribution for all individuals from 6 months to 80 years of age, and incorporated CYP2E1 distribution data (see Supplemental Material, “Structure of Human Physiological Distributions”). Supplemental Material, Figures S3A, S3B, and S3C, respectively, depict the age distributions, age-specific sex distribution, and body weight distribution used in this analysis.

**Figure 1:**
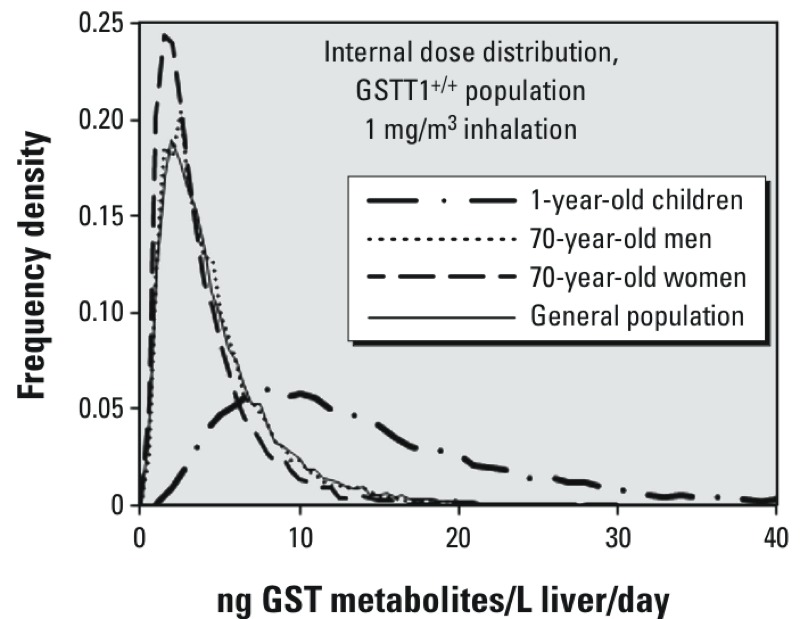
Plots of the population fraction (*y*-axis) predicted to receive various levels of the liver-specific dose of GST metabolism (ng GST metabolites/L liver/day) for the general population (0.5- to 80-year-old males and females), 1-year-old children, 70-year-old men, and 70-year-old women, within the population of GSTT1^+/+^ genotypes, given a continuous inhalation exposure to 1 mg/m^3^ dichloromethane.

The PBPK model was then used to integrate the information on various physiological and metabolic distributions to predict distributions for several internal dose metrics in the population as a whole as well as some population segments. Resulting internal dose distributions for GST-mediated metabolism due to inhalation exposure to 1 mg/m^3^ dichloromethane, corresponding to the presumed carcinogenic mode of action, are shown in Figure 1. The distributions for 70-year-old men and women are shown to differ very little from the population as a whole, although 70-year-old women are less likely to receive doses in the higher end of the range. For example, the frequency for doses between 9.5 and 10 ng GST metabolites/L liver/day is predicted to be 0.024 in the general population and 70-year-old men, but 0.014 in 70-year-old women. In contrast, the distribution for 1-year-old children is shown to be significantly broader than the population as a whole, with a larger proportion receiving higher doses. For example, 60% of 1-year-olds, but only 8.5% of the general population, are predicted to receive doses > 10 ng/L liver/day. Young children in particular are expected to receive a higher internal dose because the respiration rate per kilogram body weight is much higher for them than for adults. The much wider distribution width for 1-year-olds compared with adults is thought to occur because with high respiration rates other physiological and metabolic parameters become more rate-limiting, so the shape of the dose distribution will depend on their underlying distributions.

*Development of cancer risk values based on sensitive genotype*. As described above, the capacity for GST metabolism varies in the human population according to the presence of a relatively common “null” allele: the homozygous GSTT1 null (–/–) and active (+/+) genotypes are found in approximately 20% and 32%, respectively, of the U.S. population (Haber et al. 2002), with the heterozygous genotype representing 48% of the population. The hypothesized carcinogenic mode of action identifies metabolism by GSTT1 as a causative step, and the cancer risk is presumed to vary in direct proportion to the rate of GSTT1 metabolism. Although this rate also depends on other physiological and biochemical factors, the risk for GSTT1^+/+^ individuals will be roughly twice that for GSTT1^+/–^ individuals, whereas GSTT1^–/–^ individuals are expected to have zero risk (Warholm et al. 1994). The U.S. EPA IRIS assessment of dichloromethane derived a cancer risk estimate specifically for the presumed susceptible (high-risk) genotype by addressing the variability in GST activity through the PBPK modeling. To calculate the human cancer risk for the sensitive subpopulation (GSTT1^+/+^), the human GSTT1 activity in the PBPK model was sampled from the +/+ group distribution. The estimated GSTT1 activity for this sensitive subpopulation was then used with the cancer risk per internal dose obtained from modeling the animal cancer data to estimate the human cancer risk.

*Issues and future research pertaining to model structure*. The additional model feature that has had the greatest influence on subsequent modeling is the description of CO kinetics by Gargas et al. (1986) and Andersen et al. (1991). Because CO is produced from oxidative metabolism, tracking this metabolite should allow for a more accurate description of the split between oxidative and conjugative metabolism. Hence, inclusion of the CO model is critical in allaying model uncertainty, because it assures that the amount of metabolism predicted to occur via the saturable pathway is compared with a metabolic product specific to that pathway.

However, the CO model and subsequent comparison to observed CO levels depends on an assumed 1:1 stoichiometric conversion of dichloromethane to CO via this pathway, as was done by Gargas et al. (1986), or fitting a yield factor as was done by Andersen et al. (1991), and assuming 100% bioavailability of CO in the blood. This assumption is not supported, however, by the study of Cho et al. (2001), in which a significant distribution of CO into the limb tissue (muscle) was seen using an isolated rat hind-limb perfusion of CO (fractional recovery of only 45%). Therefore, the observations of CO produced from dichloromethane metabolism put only a lower bound on the amount of metabolism occurring via the oxidative pathway. It is possible that a greater fraction of dichloromethane metabolism is CYP-mediated than the current model predicts; the rate constant currently associated with that pathway is determined only by indirect observation of total dichloromethane pharmacokinetics with a given model structure.

Various experiments could be considered to help address this uncertainty; the first and likely simplest is a reexamination of the oxidative metabolism *in vitro*, but with concentrations spanning submicromolar to millimolar quantities. This span is needed to resolve the discrepancy in the values for *K*_m_ for the oxidative pathway, with values ranging from < 10 μM (micromolar) based on fitting the model structure to empirical pharmacokinetic data to approximately 1 mM based on *in vitro* studies (Reitz et al. 1989). The low end of the range would support or refute the assignment of the *in vivo* μM *K*_m_ to CYP metabolism that occurs with the current PBPK model, and the high end of the range could confirm the observations of Reitz et al. (1989), which might be associated with a second CYP also being active, or possibly a second binding site on CYP2E1 as suggested by Evans and Caldwell (2010). This difference of two orders of magnitude has never been adequately resolved, to our knowledge, and it calls into question the assumption that all metabolism associated with the empirically fitted linear pathway is GSH conjugation (to carcinogenic metabolites), and the remaining metabolism via the fitted saturable pathway is not.

## Conclusion

In summary, the IRIS *Toxicological Review of Dichloromethane* (U.S. EPA 2011) developed noncancer reference dose values for oral and inhalation exposures from the currently available data based on hepatotoxicity in rats and mice; the potential for neurologic effects, including long-term effects lasting after cessation of chronic exposure, is also a concern. Dichloromethane was classified as “likely to be carcinogenic to humans,” based primarily on evidence of carcinogenicity at two sites (liver and lung) in male and female B6C3F_1_ mice (inhalation exposure) and at one site (liver) in male B6C3F_1_ mice (drinking-water exposure), and supported by an association between occupational exposures and brain, liver, and hematopoietic cancers in humans. Additional epidemiological and mechanistic research pertaining to lympho-hematopoietic cancers, including studies examining genetic variability in response to specific solvent exposures, could add to our understanding of the carcinogenic potential of dichloromethane. Although there are gaps in the database for dichloromethane genotoxicity (i.e., DNA adduct formation and gene mutations in target tissues *in vivo*), a mutagenic mode of action was determined for carcinogenesis based on the available evidence of chromosomal aberrations, micronuclei, and DNA damage *in vitro* and *in vivo* that correlated with tissue and/or species availability of functional GST metabolic activity, the key activation pathway for dichloromethane-induced cancer. GSTT1 expression has been detected, at low levels, in a variety of sites in addition to liver and lung, including mammary and brain tissue (Juronen et al. 1996; Lehmann and Wagner 2008). The PBPK approach used to estimate cancer risk in the dichloromethane assessment represents some significant advancements, for example through the focus on a specific high risk genotype (GSTT1^+/+^, representing approximately 30% of the U.S. population), and incorporation of human physiological distributions for all individuals from 6 months to 80 years of age. Additional research addressing the prediction of the fraction of metabolism that occurs by the CYP versus GST pathways could address uncertainties in what is considered at this time to be the best available PBPK model, which is used as the foundation for the risk quantification.

## Supplemental Material

(266 KB) PDF
