# Supplementary material for: Human Health Effects of Dichloromethane: Key Findings and Scientific Issues
Source: Environ Health Perspect. 2014 Oct 17;123(2):114–9. doi: 10.1289/ehp.1308030 (PMC4314245; doi:10.1289/ehp.1308030)
Supplement: (266 KB) PDF [file ehp.1308030.s001.508.pdf]

Supplemental Material

Human Health Effects of Dichloromethane: Key Findings and Scientific Issues

Paul M. Schlosser, Ambuja S. Bale, Catherine F. Gibbons, Amina Wilkins, and Glinda S. Cooper

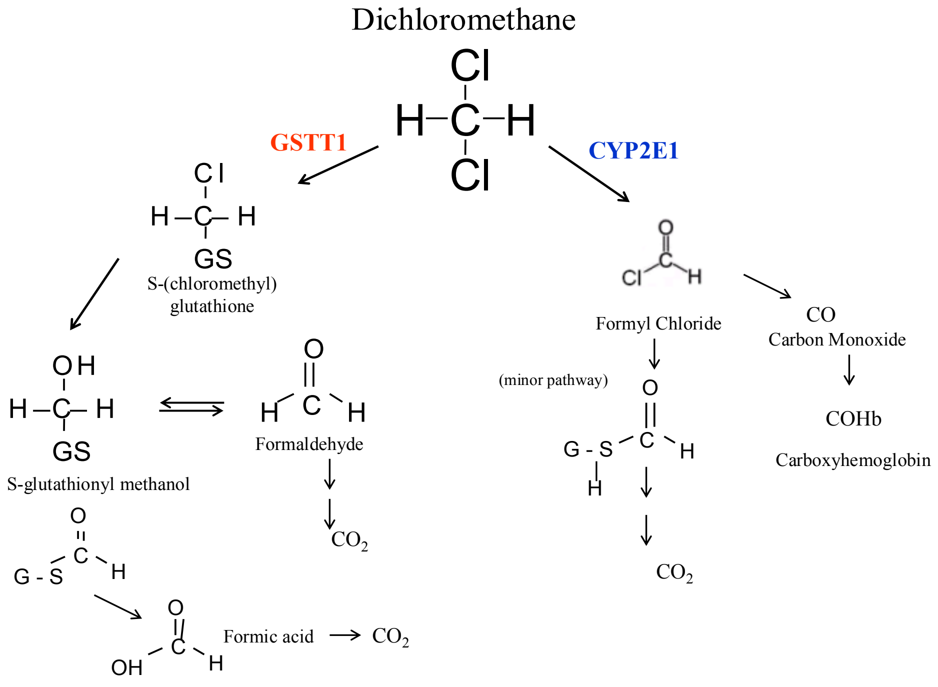

Figure S1. Proposed pathways for dichloromethane metabolism.

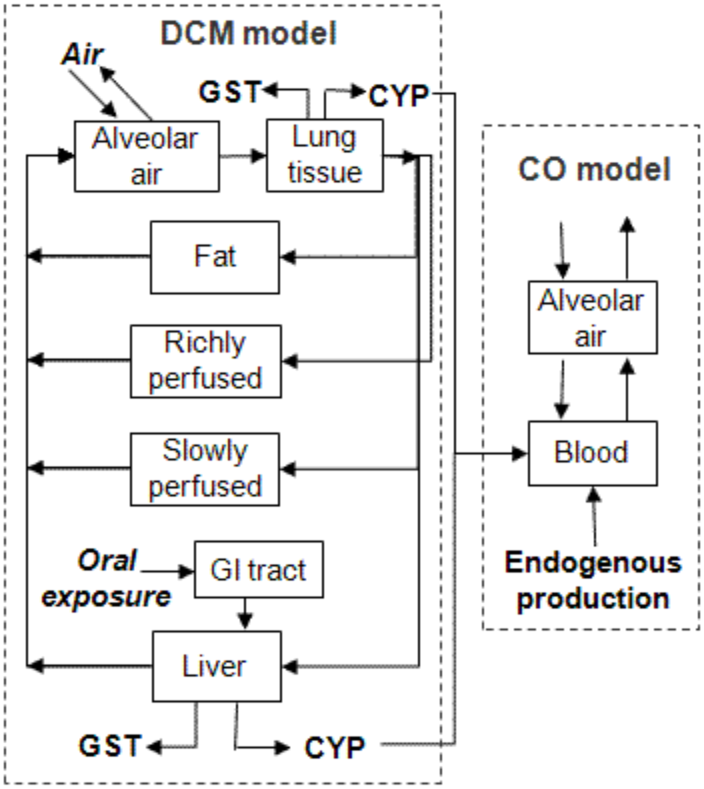

Figure S2. Schematic of PBPK model for dichloromethane (DCM) in rats, mice, and humans.

Structure of Human Physiological Distributions

The human physiological distributions were structured as follows: 1) The individual's age can either be specified (to determine age-specific risk) or selected from a distribution based on data from the United States Census (U.S. Census Bureau, 2000) (Figure S3A). 2) The individual's gender can then be either specified or selected from the age-specific distribution, again based on U.S. census data (Figure S3B); 3) The individual's body weight was then randomly generated based on age- and gender-specific functions for the mean and standard deviation, and age-specific data were used for the mean value of body-weight-normalized respiration, fat fraction, and liver fraction; the data for mean respiration and fat fraction were also gender-specific; age- (but not gender-) specific data for variance in respiration rate were also used; mean cardiac output was set as a known function of respiration rate; where other data were not available, variances were taken from David et al. (2006); and the individual's specific physiological parameters randomly generated. Total tissue fraction was then re-normalized to be 92.15% of total body weight, the remainder assumed to be teeth, bones, and hair. For CYP2E1, the scaling with body weight was evaluated using age-specific data and found to shift somewhat between individuals under 18 years of age and older individuals; the variance did not appear to be age-specific, though, and was fixed based on the result of Lipscomb et al. (2003).

The overall distribution of body weights, sampling these demographic distributions taken from different data sources (Portier et al. 2007; U.S. Census Bureau 2000), provided an interesting and surprising result that the U.S. population is bimodal (Figure S3C). The large, primary mode centered on ~ 80 kg is simply the distribution among adults, who compromise ~ 75% of the population. But while the relative rate of growth in the very young children may be fast (i.e., the

time it takes a child's weight to increase by 20%), their absolute growth rate is slower until they reach about 15 kg (33 lb). Thus an individual spends a larger fraction of his or her lifetime between 4 and 20 kg than between 20 and 36 kg (the next 16 kg) or between 36 and 52 kg.

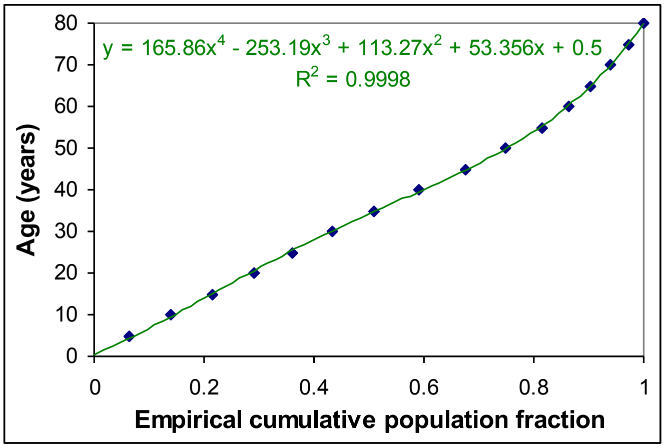

Figure S3A. U.S. age distribution, 6 months to 80 years. The cumulative fraction of the population below a given age is plotted on the x-axis vs. age in years on the y-axis. Points are values from U.S. Census Bureau (2000), in 5-year increments, normalized to the total population between 6 months and 80 years. A polynomial curve (equation shown in plot) was fit to the data. To identify a random individual in the range, a random number between 0 and 1 is selected and entered into the equation. The result is the person's age.

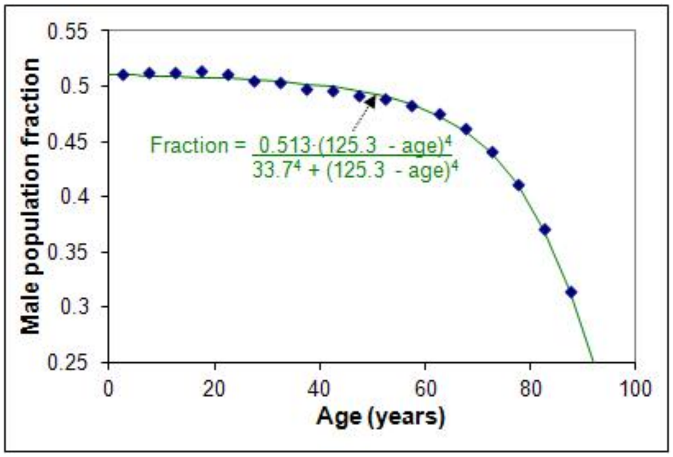

Figure S3B. U.S. age-specific gender distribution**.** The fraction of the population identified as males (y-axis) is plotted against age in years (y-axis). Points are data from the U.S. Census Bureau (2000) and the curve is an empirical function fit to the points. The solid line is an empirical equation fitted to the data (equation on plot). To identify the gender for a random individual of a given age, generate a random number, *r* between 0 and 1. If *r* is less than Fraction (age), using the equation above, the individual is male, otherwise female.

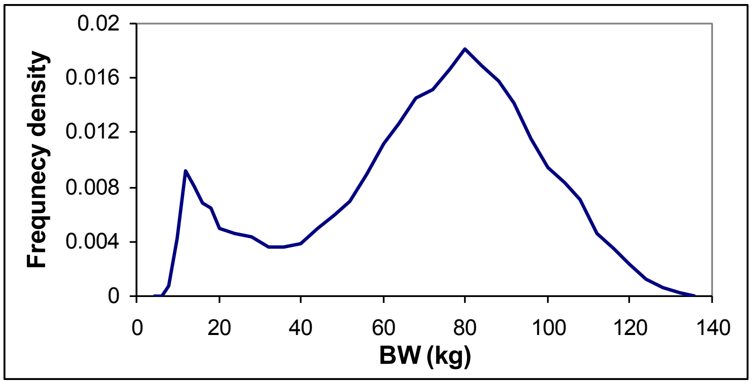

Figure S3C. Example body weight histogram from Monte Carlo simulation for 0.5–80-year-old males and females in the United States (simulated n = 10,000). Age and gender distributions are first sampled from U.S. Census Bureau (2000) data, as indicated in Figures S3A and S3B. Then body weights are sampled from assumed normal distributions given the age- and gender-specific mean and standard deviation from values reported by Portier et al. (2007) for the U.S., based on data from the National Health and Nutrition Examination Survey IV (1999-2002).

References

David RM, Clewell HJ, Gentry PR, Covington TR, Morgott DA, Marino DJ. 2006. Revised assessment of cancer risk to dichloromethane II. Application of probabilistic methods to cancer risk determinations. Regul Toxicol Pharmacol 45:55-65.

Lipscomb JC, Teuschler LK, Swartout J, Popken D, Cox T, Kedderis GL. 2003. The impact of cytochrome P450 2E1-dependent metabolic variance on a risk-relevant pharmacokinetic outcome in humans. Risk Anal 23:1221-1238.

Portier K, Tolson JK, Roberts SM. 2007. Body weight distributions for risk assessment. Risk Anal 27:11-26.

U.S. Census Bureau. 2000. QT-P1 - Age Groups and Sex: 2000, in Census 2000 Summary File 2 (SF 2) 100-Percent Data. http://factfinder2.census.gov/faces/tableservices/jsf/pages/productview.xhtml?pid=DEC_00_SF2_QTP1&prodType=table. U.S. Department of Commerce, Washington, DC.
